# Wound Healing Activities and Potential of Selected African Medicinal Plants and Their Synthesized Biogenic Nanoparticles

**DOI:** 10.3390/plants10122635

**Published:** 2021-11-30

**Authors:** Caroline Tyavambiza, Phumuzile Dube, Mediline Goboza, Samantha Meyer, Abram Madimabe Madiehe, Mervin Meyer

**Affiliations:** 1 Phytotherapy Research Group, Department of Biomedical Sciences, Cape Peninsula University of Technology, P.O. Box 1906, Bellville, Cape Town 7535, South Africa; carolinetyavambiza@gmail.com (C.T.); meyers@cput.ac.za (S.M.); 2 DSI/Mintek Nanotechnology Innovation Centre (NIC), Biolabels Node, Department of Biotechnology, University of the Western Cape, Private Bag X17, Bellville, Cape Town 7535, South Africa; phumuedube@gmail.com (P.D.); 3990471@myuwc.ac.za (M.G.); amadiehe@uwc.ac.za (A.M.M.); 3 Nanobiotechnology Research Group, Department of Biotechnology, University of the Western Cape, Private Bag X17, Bellville, Cape Town 7535, South Africa

**Keywords:** African medicinal plants, biogenic nanoparticles, wound healing, antibacterial, antioxidant, anti-inflammatory

## Abstract

In Africa, medicinal plants have been traditionally used as a source of medicine for centuries. To date, African medicinal plants continue to play a significant role in the treatment of wounds. Chronic wounds are associated with severe healthcare and socio-economic burdens despite the use of conventional therapies. Emergence of novel wound healing strategies using medicinal plants in conjunction with nanotechnology has the potential to develop efficacious wound healing therapeutics with enhanced wound repair mechanisms. This review identified African medicinal plants and biogenic nanoparticles used to promote wound healing through various mechanisms including improved wound contraction and epithelialization as well as antibacterial, antioxidant and anti-inflammatory activities. To achieve this, electronic databases such as PubMed, Scifinder^®^ and Google Scholar were used to search for medicinal plants used by the African populace that were scientifically evaluated for their wound healing activities in both in vitro and in vivo models from 2004 to 2021. Additionally, data on the wound healing mechanisms of biogenic nanoparticles synthesized using African medicinal plants is included herein. The continued scientific evaluation of wound healing African medicinal plants and the development of novel nanomaterials using these plants is imperative in a bid to alleviate the detrimental effects of chronic wounds.

## 1. Introduction

### 1.1. Wounds

Wounds are defined as injury to living tissue which leads to the disruption of its normal anatomical structure and function [1]. They arise due to physical, chemical, thermal, microbial or immunological damage to the tissue [2]. Regardless of the aetiology and type, wounds can cause damage to the tissue and disrupt the surrounding environment. The damage can affect the integrity of the skin epithelial layer and can also extend into the subcutaneous tissue disrupting other structures such as tendons, muscles and nerves [1,3]. Failure of wounds to heal normally leads to chronic wounds. Chronic wounds are a silent epidemic claiming the lives of many individuals worldwide. In the past decade, it has been estimated that 6 million people suffer from chronic wounds worldwide [2,4,5,6]. Wounds have a significant negative impact on the economic and social lives of patients and their families. They cause severe pain, physical disability such as immobility and loss of function, loss of self-esteem, depression and anxiety as well as premature death [7,8]. Not only do wounds affect the patients’ social life, they also pose a great financial burden on patients and the healthcare systems. Chronic wounds consume a great amount of healthcare resources around the world [8].

Chronic wounds are predominantly a condition of the elderly, however, their prevalence is expected to increase in all age groups because of the increase in conditions that impede wound healing, such as diabetes, obesity and vascular disorders [9,10]. The high and increasing prevalence of these conditions continues to increase the global burden of chronic wounds. Unfortunately, to our knowledge, there are no statistical records on the prevalence of chronic wounds in Africa.

### 1.2. Classification of Wounds

Wounds can be classified depending on their healing time. Acute wounds heal timely, following the normal wound healing process. They result in the restoration of anatomical and functional integrity of the normal tissue [11]. On the other hand, chronic wounds require prolonged time to heal, and they do not progress through the normal stages of wound healing [12]. Given their complexity, chronic wounds tend to fall into four categories, namely pressure ulcers, venous ulcers, diabetic ulcers and arterial insufficiency ulcers [8,13].

Wounds can also be classified as open (those associated with disruption and discontinuity of the skin) or closed (when there is damage to the underlying tissue but the skin is intact) [12]. Open wounds can be further classified as incisions (cut by a sharp object such as a scalpel), lacerations (tear like wounds), abrasions (scraping of the outer skin layer), puncture wounds, penetration wounds (caused by an object entering or exiting the skin surface) and gunshot wounds. Most open wounds are often associated with a wide range of bacterial and fungal infections as the underlying tissues are exposed to the outside environment [5,12]. The challenge of antimicrobial resistance in treating these infections increases the complications and burden of these wounds [14,15]. Repetitive trauma, which is common in diabetic foot ulcers, can also delay or even stop the wound healing process [16]. Closed wounds include contusions (caused by a blunt force trauma that damages tissue under the skin), haematomas (accumulation of blood under skin due to a damaged blood vessel) and crush injuries (occurs when great external force is applied on the skin over a long period of time) [17].

### 1.3. Phases of Wound Healing

Wound healing is a complex physiological process which aims to restore the anatomical structure and function of an injured tissue [18]). This process is divided into four integrated phases, namely haemostasis, inflammation, proliferation and tissue remodelling. Haemostasis involves vascular constriction and coagulation. Platelets attach to exposed collagen surfaces and extracellular matrix leading to the activation of the coagulation cascade and clot formation, thereby preventing blood loss [19,20]. This process is mediated by growth factors released by platelets such as Epidermal growth factor (EGF), Platelet-derived growth factor (PDGF), Transforming growth factor-beta (TGF-β) and Fibroblast growth factor (FGF). These growth factors together with some cytokines play a vital role in the movement of monocytes and neutrophils to the wound site initiating the inflammatory phase [5,21]. During inflammation, neutrophils and monocytes penetrate the wound site. Neutrophils, which are the first leukocytes to infiltrate the wound site, eliminate invading microorganisms through phagocytosis and release reactive oxygen species and proteolytic enzymes [22,23]. Neutrophils also release IL-8 aiding chemotaxis which attracts monocytes and other cells to the wound site [19]. Monocytes then differentiate into macrophages which continue the process of phagocytosis and cleansing of the wound. They also initiate the development of granulation tissue and angiogenesis by releasing various growth (FGF, EGF, TGF-b, and PDGF) and cytokines (IL-1 and IL-6) [24,25].

The proliferation phase comes immediately after the cleansing of the wound and then the repair process (building of new tissue). This phase is characterized by the formation of granulation tissue, angiogenesis, wound contraction and epithelialization [26]. It involves the activity of many cells including fibroblasts, keratinocytes, and endothelial cells. Granulation involves the deposition of newly synthesized extracellular matrix composed of collagen, fibrin and fibronectin which replaces the damaged tissue [27]. Angiogenesis by endothelial cells ensures sufficient supply of nutrients and oxygen to the granulation tissue [28]. Fibroblasts and myofibroblasts cause the wound to contract by pulling the wound edges together. Subsequently keratinocytes migrate over the granulation tissue from the wound edges towards the center to cover the wound and protect the underlying issue [21,29]. The tissue remodeling phase (last phase) occurs after the wound has closed. This phase aims to achieve maximum tensile strength and flexibility of the new tissue. However, the wound never achieves the same level of tensile strength as the normal tissue. Remodeling and reorganization of collagen fibres occurs, resulting in the formation of cross-linkages which reduces scar thickness. The level of vascularity decreases as the scar matures [30,31]. The cells playing a part in wound repair are removed by apoptosis as they are no longer needed [32].

### 1.4. Risk Factors and Conditions Associated with Wound Healing

The wound healing process is not only complex but also fragile, as it is susceptible to numerous interruptions and failure. Various factors can affect one or more phases of wound healing as well as its sequence and time frame, leading to the impairment of this process. These factors delay wound healing resulting in poor outcomes thereby increasing the patient morbidity and mortality [26]. The risk factors of wound healing are classified into local and systemic. Systemic factors affect the general health of an individual, which influences their ability to heal normally. These factors include age, stress, chronic diseases (such as diabetes mellitus, hepatic and renal failure), obesity, alcoholism, medication and immunocompromising conditions (such as cancer and AIDS) [15]. Local factors are those that directly influence the characteristics of the wound itself. These include infection, oxygenation, venous insufficiency and the presence of foreign material in the wound. All these factors are related, usually systemic factors act through the local factors in affecting wound healing [33].

Infections are one of the most common complications of wounds, as they disrupt the normal wound healing process, leading to the development of chronic wounds. Frequently isolated bacterial species in wounds include *Staphylococcus aureus*, *Pseudomonas aeruginosa* and *β-hemolytic streptococci* [15,34]. The loss of skin integrity as a result of the injury enables pathogens to invade the wound site and cause infections. Bacterial invasion affects all stages of wound healing, particularly the inflammatory phase. This phase plays an important role in microbial clearance and wound cleansing. However, in the presence of an infection, inflammation is prolonged due to the increased microbial load and incomplete microbial clearance [35]. Bacterial toxins decrease chemotaxis and increase the secretion of pro-inflammatory cytokines such as IL-1 and TNF-α. This further prolongs the inflammation, causing the wound to enter a chronic state [25,34]). The continuous inflammatory phase increases the presence of neutrophils, which will lead to the degradation of the extracellular matrix and loss of important wound healing growth factors (TGF-β and PDGF) [36]. The increase in neutrophils will also increase the production of reaction oxygen species (ROS). The ROS are essential in cellular metabolism, however, when produced in excess they cause cellular damage and further affect the healing of the wound [11]. In the treatment of wounds, it is therefore imperative for a wound healing agent to have antimicrobial, anti-inflammatory and antioxidant activities as this will aid in the healing of the wound.

### 1.5. Conventional Treatment and Management of Wounds

The care and management of wounds is of great importance in wound healing, as it ensures and aids proper wound healing. The aim of good wound management is to provide and maintain a warm, moist, non-toxic environment which supports natural wound healing. Poorly managed wounds can lead to the development of non-healing chronic wounds [37]. Hygiene is one of the important factors in wound management as it minimizes the risk of wound infections. Wounds undergo debridement before the application of topical treatments such as antimicrobial and silver wound dressings, silver sulfadiazine creams and oils and sprays. Debridement is the removal of necrotic tissue from the affected area in-order to promote wound healing [20]. Topical therapies are the first line of treatment of wounds. After assessment, and if healing is not achieved, advanced wound therapies are then used. The most common advanced wound therapies include negative pressure wound therapy, administration of growth factors, hyperbaric oxygen and skin grafts [8].

These therapies are very costly, hence, not all individuals can access them, especially in poor resource countries. They are also associated with severe complications such as bleeding, infection, barotrauma and oncogenesis [38,39,40]. Although these therapies are available, the burden of chronic wounds is increasing. The search for new affordable and easily accessible wound treatment drugs and therapies is therefore vital. One reliable source of drug discovery is medicinal plants as they have been used traditionally for many years in the treatment of many human ailments. It has been reported that 25% of modern drugs were derived from plants [41,42]. Nanomaterials synthesized from medicinal plants were also shown to be beneficial in wound healing. In this article, we will review different wound healing mechanisms of African medicinal plants as well as those of biogenic nanoparticles synthesized using African medicinal plants. To achieve this, electronic databases such as PubMed, Scifinder^®^ and Google Scholar were used in the search. The African medicinal plants and their biogenic nanoparticles were evaluated for their wound healing activities in both in vitro and in vivo models from 2004 to 2021.

## 2. African Medicinal Plants in the Treatment of Wounds

The African continent is well known for an immense biodiversity distribution with nearly 45,000 different plant species of which about 5000 species have been used as traditional medicines [5]. Throughout history, medicinal plants have been used exclusively in the treatment and management of different diseases, especially in poor communities. It is documented that 80% of the African populace rely on medicinal plants as their primary source of medication as medicinal plants are more affordable, easily accessible and are associated with fewer side effects when compared to conventional medicines [43]. Medicinal plants are a good source of compounds which could serve as leads for drug discovery for wound healing [44]. The wound healing activities displayed by medicinal plants are attributed to the presence of bioactive chemicals such as phenols, alkaloids, triterpenes, and flavonoids. In wound healing, these bioactive compounds have been reported to have antioxidant and antimicrobial activities, improve collagen deposition and increase the proliferation of both fibroblasts and keratinocytes [5,45]. In most instances, the wound healing process progresses naturally. However, the presence of an infection can cripple the healing process by lengthening the inflammatory phase, disturbing the normal clotting mechanisms, disrupting leukocyte function and angiogenesis [46]. Thus, the antimicrobial effects exhibited by many plants explain the significant roles they display in the healing of wounds. On the other hand, phenolic compounds were reported to play an important role in the healing of wounds because of their antioxidant effect against free radicals which negatively influence the progression of wound healing [47]. Various reports have provided information on plants used in Africa for the treatment of wounds. Some of these plants are listed in Table 1 below.

## 3. Nanotechnology as a Wound Healing Intervention

Medicinal plants have been incorporated in the field of nanotechnology in a bid to enhance the activity of the plants. Different nanoparticles including silver [63,70,74,75], gold [76,77], titanium dioxide [78] and selenium [79,80] have been successfully synthesized using plants.

Silver containing wound therapies have a long history of use in the treatment of chronic wounds. Silver releasing agents are known to inhibit the manifestation of a variety of microorganisms including fungi, Gram-positive and Gram-negative bacteria on wounds [81]. This is due to silver interfering with the electron transport system or binding to and inhibiting DNA replication of the microbes [82]. Additionally, these silver therapeutics display low systemic toxicity as they can be neutralized by anions in body fluids. Some of the most widely used silver based wound dressings, such as Askina Calgitrol Ag^®^, Aquacel^®^ Ag Dressing, 3M Tegaderm Alginate Ag Silver Dressing and Systagenix Silvercel antimicrobial alginate dressing, contain silver alginates, whilst common topical agents such as Silvadene, Thermazene^®^ and SSD Cream^®^ contain silver sulfadiazine [83,84,85,86,87,88]. Certain dressings like Aquacel^®^ Ag Dressing may cause skin irritation and allergies [86]. The more extensive methods of chronic wound treatment such as negative pressure wound therapy, administration of growth factors, hyperbaric oxygen and skin grafts have been reported to be costly, unsafe and detrimental to surrounding healthy tissues [39]. Hence, novel wound healing strategies with increased efficacy and limited side effects are constantly being developed. In this line, nanotechnology has the potential to address these matters of concern, as it is providing novel materials that exhibit enhanced size-dependent properties [27,89,90].

Nanotechnology has played a notable role in the delivery of improved methods for disease diagnostics and therapeutics. To date, about 51 nanopharmaceuticals synthesized from liposomes, polymers, nanocrystals, proteins, micelles and inorganic reducing agents have been approved by the US Federal Drug Agency and are available in clinical practices, whilst several nanomedicines are undergoing clinical trials [91,92]. Examples include the widely used Acticoat^TM^ Flex 3 and Acticoat©, which are silver nanoparticle-based products that promote wound healing whilst reducing pain and infection at the wound site [93,94]. Silver nanoparticles have been shown to promote wound healing with improved cosmetic appearance. The activity has been attributed to their antimicrobial properties, reduction in wound inflammation and modulation of fibro-genic cytokines. Tian et al. (2007) reported that silver nanoparticles had an increased healing rate compared to silver sulfadiazine, the standard treatment for burn wounds [95]. Despite their popularity, the prolonged use of these silver based wound therapies may slow the wound healing process due to epithelial and fibroblast cytotoxicity and cause negative cosmetic effects such as argyria. Other nanoparticles such as gold, zinc oxide and selenium have also been used to promote wound healing [96,97]. Thermo-responsive gels containing gold nanoparticles displayed antimicrobial and wound healing properties as shown by in vitro, in vivo and histopathological assays [98]. Low concentrations of gold nanoparticles, 34 nm in size, significantly promoted the proliferation of keratinocytes, which are important in wound closure [99]. This proved the great efficiency and potential of the gold nanoparticles as wound therapeutics and a probable alternative to autografts.

### 3.1. Biogenic Nanoparticles in Wound Healing

Although several chemical and physical synthesis methods for the production of nanoparticles are known and used, the synthesis of nanoparticles using biological models has several advantages over these methods. This is due to the fact that nanoparticle synthesis using physio-chemical approaches utilizes organic solvents and toxic reducing agents whilst producing hazardous waste-products [100]. The biologically synthesized nanoparticles possess a great advantage where applicability in industrial and human health care products is concerned. Numerous biological approaches which employ different natural resources such as plants, microorganisms and algae in the synthesis of nanoparticles are available [77]. Although microorganisms are widely used to reduce metallic salt solutions to form corresponding nanoparticles, the process uses toxic and costly reactants that result in an unsuitable platform for biocompatible synthesis [27]. On the other hand, plants are readily used to synthesize nanoparticles as they are easily accessible and can promote rapid nanoparticle synthesis [101]. Bioactive plant phytochemicals (alkaloids, phenolic acids, polyphenols, saponins, proteins, sugars, and terpenoids) are believed to have significant roles in the synthesis of nanoparticles [102]. They are involved in the reduction of metallic ions and also act as capping agents that stabilize the synthesized nanoparticles [103].

The unique features of synthesized nanoparticles such as large surface area to volume ratio allow them to bind and deliver small-sized beneficial compounds to target sites. The ability of biogenic nanoparticles to combine with bioactive drugs and other useful compounds can result in a synergistic effect, which further enhances their efficiency and effectiveness. Combining *Urtica dioica* silver nanoparticles and the antibiotics Amoxicillin, Amikacin, Kanamycin, Tetracycline and Cefotaxime showed a synergistic inhibitory activity against *S. aureus*, *Staphylococcus epidermidis*, *Escherichia coli*, *Klebsiella pneumoniae*, *Bacillus cereus*, *Bacillus subtilis*, *Serratia marcescens* and *Salmonella typhimurium.* This was shown by a 0.1- to 17.8-fold increase in the zones of inhibition [104]. Many polymers including chitosan, hyaluronan, pyridoxine and collagen have been combined with silver nanoparticles to form silver nanocomposites with enhanced wound healing properties [97]. The combination of silver nanoparticles with tetracycline was shown to significantly improve wound healing and wound infection control in Albino mice [105].

Given the history and benefits of medicinal plants found on the African continent, combining these plants and nanotechnology can lead to the development of important nanomedicines. Though some of the medicinal plants are not native to the African continent, several are found and harvested in one or more African countries. The bio-synthesized nanoparticles may potentially improve wound healing efficacy, exhibit better antimicrobial activity and/or promote accelerated wound closure compared to the raw materials and current treatments as shown in Table 2. The enhanced activity could be attributed to the expected increase in the stability of bioactive phytochemicals within the nanoparticles and the large surface area of the synthesized nanoparticles [75,106,107].

### 3.2. Biological Nanoparticles with Potential Wound Healing Properties

*Delonix elata* copper oxide nanoparticles (36 nm in size) applied on human wounds after anal rectum surgery caused rapid wound epithelialization [117]. Nanoparticle treated wounds also showed a weaker inflammatory response probably resulting from cytokine modulation. An association between wound healing and the anti-inflammatory and/or antioxidant activities of therapeutics has been established. Anti-inflammatory agents are important in controlling inflammation whilst antioxidants address oxidative stress at the site of the wound thereby accelerating wound healing [118]. Both these properties have also been reported to decrease the production of pus at the wound site. Numerous nanoparticles synthesized using medicinal plants found on the African continent have been shown to exhibit either antioxidant and/or anti-inflammatory activity (Table 3), classifying them as potential wound healing therapies.

The potential of biogenic nanoparticles, especially those synthesized using African medicinal plants has not been fully explored. In many instances biogenic nanomaterials have been proven effective for most biomedical applications. Numerous nano-based medicines have been approved by the FDA for use while many others are on trial. It is therefore of great importance to investigate all wound healing activities of biogenic nanoparticles as this might lead to the discovery of efficacious wound healing therapeutics.

## 4. Conclusions and Recommendations

A thorough and effective wound healing process is crucial to the well-being and socio-economic state of patients. The complexity of a typical healing process is due to the various components involved at both cellular and subcellular levels. Although classic topical wound therapeutics including medicinal plants and silver sulfadiazine are still common among the African populace, the prevalence of chronic wounds remains high. Henceforth, current technologies such as nanotechnology should focus mainly on the molecular and cellular aspects of wound healing in the development of therapeutic interventions. Considering that silver nanoparticle impregnated wound dressings are slowly becoming a standard in wound and burn therapy, the discovery of the full potential of nanomedicine is necessary. Unlike conventional physio-chemical methods, the green synthesis of nanoparticles using known medicinal plants is a non-toxic approach in the production of biocompatible nanoparticles. It is also cost effective, making it affordable for low resource African countries to utilize these strategies to develop alternative treatments. Given the great potential of biogenic nanoparticles in various biomedical applications and medicinal plants in drug discovery, it is imperative that widely used wound healing medicinal plants native to the African continent be incorporated in nanomaterials. However, the scarcity of scientific evidence reporting the wound healing activities of biogenic nanoparticles synthesized using these African medicinal plants is concerning. Green nanotechnology still has a vast selection of raw materials still to be explored on the African continent. Therefore, the development of novel nanomaterials exhibiting impressive wound healing activity and the further understanding of the mechanisms underlying their beneficial properties is important for future research.

## Figures and Tables

**Table 1:** Wound healing activities of selected African medicinal plants.

Plant Name	Family	Plant Part Used	Mode of Wound Healing	Reference
*Acacia senegal*	*Mimosae*	Root	Antimicrobial	[48]
*Aloe ferox*	*Asphodelaceae*	Leaf gel	Antioxidant, anti-inflammatory and antibacterial activities	[49,50]
*Aloe vera*	*Asphodelaceae*	Leaf gel	Enhance mature granulation, antioxidant, anti-inflammatory and antibacterial activities	[45]
*Aspalathus linearis*	*Fabaceae*	Leaves	Antioxidant, anti-inflammatory and antibacterial activities	[51]
*Argyreia nervosa*	*Convolvulaceae*	Leaves and roots	Antibacterial and anti-inflammatory	[52,53]
*Boerhavia diffusa*	*Nyctaginaceae*	Whole plants	Increased myoblast migration, antioxidant	[44]
*Boophone disticha*	*Amaryllidaceae*	Bulb	Antimicrobial, anti-inflammatory	[53,54]
*Bridelia ferruginea*	*Combretaceae*	Leaf	Stimulation of fibroblasts	[55]
*Bulbine frutescens*	*Asphodelaceae*	Gel sap	Improvement of wound contraction	[56]
*Catharanthus roseus*	*Apocynaceae*	Leaf	Improved wound contraction, decreased epithelization period and antibacterial activities	[57]
*Centella asiatica*	*Umbelliferae*	Leaves	Increased cellular proliferation, angiogensis collagen synthesis, antioxidant and anti-inflammatory	[58,59]
*Cotyledon orbiculata*	*Crassulaceae*	Leaf gel	Antioxidant, anti-inflammatory and antibacterial activities	[60,61,62,63]
*Ficus asperifolia*	*Moraceae*	Bark	Stimulation of fibroblasts, antioxidant	[46]
*Gossypium arboreum*	*Malvaceae*	Leaf	Stimulation of fibroblasts, antioxidant	[46]
*Gunnera perpensa*	*Gunneracea*	Root	Antioxidant and antibacterial activities	[64,65]
*Gymnosporia senegalensis*	*Celestraceae*	Leaf, bark, roots	Anti-inflammatory and antimicrobial activities	[66]
*Haemanthus coccineus*	*Amaryllidaceae*	Bulb	Anti-inflammatory	[58,67]
*Maytenus heterophylla*	*Celastraceae*	Crushed leaves	Anti-inflammatory and antimicrobial activities	[68]
*Merwilla natalensis*	*Asparagaceae*	Fresh and burnt bulb scales	Anti-inflammatory and antimicrobial activities	[50]
*Ocimum gratissimum* L.	*Lamiaceae*	Leaf	Antibacterial and activities	[55,69]
*Parkia biglobosa*	*Fabaceae*	Stem	Stimulation of fibroblasts and antibacterial activity	[55]
*Sutherlandia frutescens*	*Fabaceae*	Leaves	Antibacterial	[70]
*Tecoma capensis*	*Bignoniaceae*	Bark	Improved wound contraction and re-epithelialization	[71]
*Terminalia sericea*	*Combretaceae*	Bark	Antioxidant, anti-inflammatory and antibacterial activities	[64,72]
*Vernonia amygdalina*	*Asteraceae*	Leaf	Improved wound contraction and re-epithelialization	[73]

**Table 2:** Biogenic nanoparticles synthesized using African medicinal plants exhibiting wound healing activities.

Plant Name	Nanoparticle Type	Particle Size	Experimental Outcomes	Reference
*Azadirachta indica*	Silver	60–85 nm	(a) In diabetic rats, the nanoparticles show increased wound contraction and accelerated wound healing compared to the control and the drug alone.	[108]
		33 nm	(b) Synthesized silver nanoparticles displayed enhanced antibacterial and antioxidant effects when compared to the leaf extracts alone. The nanoparticle infused PF127 hydrogel improved the wound contraction rate in mice.	[109]
		30 nm	(c) The wound beds where the nanoparticles were topically applied showed no microbial growth, haemorrhage, or formation of pus throughout treatment. The nanoparticle treated female BALB/c mice showed better wound-healing capacity when compared to control group animals.	[74]
*Cassia auriculata* L.	Silver	15–90 nm	Nanoparticles showed an increased percentage of wound contraction in both excision and incision wound models when compared to the drug Povidone iodine as well as the *C. auriculata* extracts.	[110]
*Catharunthus roseus*	Silver	20 nm	(a) When the silver nanoparticles were topically applied on open wounds, the treated animals exhibited better wound healing activity and decreased inflammation compared to those treated with the respective aqueous plant extract.	[111]
		30 nm	(b) Silver nanoparticle treated wounds on female BALB/c mice showed decreased irritation with greater wound healing capacity when compared to the positive control.	[74]
*Curcuma longa* L.	Silver	15–40 nm	Synthesized nanoparticles increased fibroblast cell proliferation and migration indicating effective wound healing.	[112]
*Falcaria vulgaris*	Silver	40–45 nm	The nanoparticles promoted the healing of cutaneous wounds on male rats by decreasing the wound area and increasing wound contracture, antimicrobial and antioxidant activity, fibroblast-fibrocyte ratio and macrophages at the wound site when compared to the activity of *F. vulgaris* extracts and tetracycline.	[113]
*Mimosa pudica*	silver	7.63 nm	Biosynthesized nanoparticles incorporated in an electrospun polyvinyl alcohol (PVA) improved keratinocyte migration when compared to the activity of PVA alone.	[114]
*Moringa oliefera*	Titanium dioxide	100 nm	The synthesized nanoparticles promoted significant wound closure in Albino rats when compared to a standard commercial wound therapeutic.	[75]
*Prosopis juliflora*	Silver	10–20 nm	The percentage of wound healing was increased for the nanoparticles when compared to that of Povidine-iodine.	[115]
*Woodfordia fruticosa*	Gold	13 nm	Topical application of the synthesized nanoparticles on excised wounds on Wistar albino rat models resulted in the effective prevention of microbial adhesion and accelerated wound closure in comparison to the activity of 5% Povidone-iodine.	[116]

**Table 3:** Biogenic nanoparticles synthesized using African medicinal plants with bioactivities that promote wound healing.

Plant Name	Nanoparticle	Size	Anti-Inflammatory Activity	Antioxidant Activity	Reference
*Allium ampeloprasum*	Silver	8 nm	-	Yes	[119]
*Aloe vera*	Selenium	7–48 nm	-	Yes	[80]
*Areca catechu*	Silver	18.2–24.3 nm	-	Yes	[120]
*Artocarpus altilis*	Silver	38 nm	-	Yes	[121]
*Cola nitida*	Silver	12–80 nm	-	Yes	[122]
*Cotyledon orbiculata*	Silver	20–60 nm	Yes	-	[63]
*Cuminum cyminum*	Silver	-	Yes	-	[123]
*Garcinia indica*	Gold	20–30 nm	-	Yes	[124]
*Garcinia indica*	Silver	5–30 nm	-	Yes	[125]
*Helicteres isora*	Silver	38.23 nm	-	Yes	[126]
*Hypoxis hemerocallidea*	Gold	27 nm	Yes	-	[127]
*Kalanchoe pinata*	Zinc oxide	24 nm	Yes	-	[128]
*Nigella sativa*	Silver	25.2 nm	Yes	Yes	[129]
*Oxalis corniculata*	Silver	30 nm	-	Yes	[130]
*Phoenix dactylifera*	Zinc sulphide	<70 nm	-	Yes	[131]
*Terminalia bellirica*	Silver	11 nm	Yes	Yes	[132]
*Terminalia bentazoe*	Silver	7 nm	Yes	Yes	[132]
*Terminalia catappa*	Silver	10 nm	Yes	Yes	[132]
*Terminalia mellueri*	Silver	10 nm	Yes	Yes	[132]
*Zingiber officinale*	Selenium	100–150 nm	-	Yes	[79]

## Data Availability

Not applicable.
